# Muscarinic Acetylcholine Receptors in the Retina—Therapeutic Implications

**DOI:** 10.3390/ijms22094989

**Published:** 2021-05-08

**Authors:** Yue Ruan, Andreas Patzak, Norbert Pfeiffer, Adrian Gericke

**Affiliations:** 1 Department of Ophthalmology, University Medical Center, Johannes Gutenberg University Mainz, Langenbeckstr. 1, 55131 Mainz, Germany; norbert.pfeiffer@unimedizin-mainz.de (N.P.); adrian.gericke@unimedizin-mainz.de (A.G.); 2 Institute of Vegetative Physiology, Charité-Universitätsmedizin Berlin, Charitéplatz 1, 10117 Berlin, Germany

**Keywords:** muscarinic acetylcholine receptors, glaucoma, retina, retinal ganglion cells, therapeutic strategy

## Abstract

Muscarinic acetylcholine receptors (mAChRs) belong to the superfamily of G-protein-coupled receptors (GPCRs). The family of mAChRs is composed of five subtypes, M_1_, M_2_, M_3_, M_4_ and M_5_, which have distinct expression patterns and functions. In the eye and its adnexa, mAChRs are widely expressed and exert multiple functions, such as modulation of tear secretion, regulation of pupil size, modulation of intraocular pressure, participation in cell-to-cell signaling and modula-tion of vascular diameter in the retina. Due to this variety of functions, it is reasonable to assume that abnormalities in mAChR signaling may contribute to the development of various ocular diseases. On the other hand, mAChRs may offer an attractive therapeutic target to treat ocular diseases. Thus far, non-subtype-selective mAChR ligands have been used in ophthalmology to treat dry eye disease, myopia and glaucoma. However, these drugs were shown to cause various side-effects. Thus, the use of subtype-selective ligands would be useful to circumvent this problem. In this review, we give an overview on the localization and on the functional role of mAChR subtypes in the eye and its adnexa with a special focus on the retina. Moreover, we describe the pathophysiological role of mAChRs in retinal diseases and discuss potential therapeutic approaches.

## 1. Introduction

In 1914, Henry Dale first observed that the actions of acetylcholine (ACh) could be divided into nicotine-like and muscarine-like effects, respectively [1]. Preganglionic parasympathetic neurons release neurotransmitters, such as acetylcholine, from synaptic vesicles of axon terminals into the synaptic cleft. From there, neurotransmitters can bind to receptors in postganglionic parasympathetic cell membranes [2]. Muscarinic acetylcholine receptors (mAChRs) are widely localized on postganglionic parasympathetic neurons and are widely expressed in the central nervous system [3]. Apart from neurons, mAChRs are expressed on many other cell types [4]. In the eye and its adnexa, mAChRs were found to be expressed in the cornea, lens, uvea, conjunctiva, sclera, retina and the lacrimal gland [5,6,7,8]. Hence, it is not surprising that mAChRs are involved in diverse important physiological functions in the eye, such as tear fluid production, goblet cell secretion, keratocyte migration and proliferation, pupil size regulation, ocular drainage, lens cell signaling and ocular growth as well as cell-to-cell signaling and vascular reactivity in the retina [9,10,11,12,13]. An increasing number of studies demonstrates that mAChRs are potential pharmacological targets for the treatment of various ocular diseases, such as glaucoma and myopia [14,15].

Glaucoma is a neurodegenerative disease, which is characterized by the impairment and loss of retinal ganglion cells (RGCs) and optic nerve fibers, which may lead to irreversible blindness [16]. It has been estimated that there are around 80 million people with glaucoma worldwide [17]. According to another estimate, the global number of people affected by glaucoma will increase to 111.8 million in 2040 [18]. Although elevated intraocular pressure is a major risk factor for glaucoma, several population-based studies reported that intraocular pressure is within the normal range in a large portion of individuals with glaucoma [19,20]. In clinical practice, lowering intraocular pressure (IOP) is essential to prevent development and progression of the disease and to preserve the patients’ quality of life [16]. However, in almost half of the patients the disease continues to progress despite normalization of IOP [21]. Hence, novel complementary retinal neuroprotection strategies would be valuable to reduce progressive neurodegeneration in the retina [22]. Pathologic myopia with characteristic degenerative changes in the sclera, choroid, and retinal pigment epithelium is a major cause of visual impairment and blindness worldwide by increasing the risk for ocular complications, such as macular degeneration, retinal detachment and glaucoma [23]. The global prevalence of pathologic myopia is rising rapidly, especially in the younger Asian population [24]. Holden et al. projected that by 2050 the number of people with myopia will increase to 4.758 billion (49.8% of the global population), and 938 million people (9.8% of the world’s population) will have high myopia [25]. There is still no effective therapy in the clinical routine to prevent the progression of pathologic myopia, which makes the disease an increasing global health concern.

MAChRs belong to the class of G-protein-coupled receptors (GPCRs) containing seven transmembrane segments, which transfer signals into the cell via coupling with G-proteins. The G-proteins modulate the activity of a number of different effectors, such as ion channels and enzymes [26,27]. The mAChR family is composed of five subtypes, M_1_, M_2_, M_3_, M_4_ and M_5_ with different molecular and signaling properties [3,28,29]. For example, M_1_, M_3_ and M_5_ have been reported to typically couple to G proteins of the Gq/11 family. However, M_2_ and M_4_ receptors have been shown to preferentially couple to G proteins of the Gi and Go family [3,29]. Some studies reported that mAChR agonists reduce IOP and exert neuroprotective effects in glaucoma [30,31,32]. The non-subtype-selective mAChR antagonist, atropine, has been shown to inhibit scleral proliferation and matrix synthesis, and to prevent axial elongation of the eyeball providing a novel therapeutic approach for myopia control [14,33]. Unfortunately, non-subtype-selective mAChR agonists may exert ocular adverse effects in clinical practice, which are related to its constricting effects on the ciliary and pupillary sphincter muscle and systemic adverse effects including increased salivation and sweating, vomiting, diarrhoea and tachycardia [34,35]. For example, the non-subtype-selective mAChR agonist, pilocarpine, which has been used for long-term IOP control can cause blurred vision, brow ache from ciliary spasm and rarely retinal detachment [35]. In addition, the non-specific mAChR antagonist, atropine, acutely induces cycloplegia and photophobia and on the long term might cause premature presbyopia, cataract, and light damage in the retina [36]. 

To design more specific mAChR-based therapeutic approaches with less side-effects, it is crucial to identify the distribution and physiological function of individual mAChR subtypes and to test the use of highly subtype-selective ligands in laboratory and clinical studies. In this review, we summarize and discuss the localization, the functional and the pathophysiological role of individual mAChR subtypes in the retina. Additionally, we discuss potential therapeutic strategies targeting individual mAChR subtypes.

The identification of literature was carried out via a search on PubMed. The PubMed database search included the following keywords: ((“muscarinic acetylcholine receptors” OR “muscarinic acetylcholine receptor subtypes” OR “muscarinic receptors” OR “mAChR” OR “mAChR subtypes” OR “M_1_ muscarinic acetylcholine receptor” OR “M_2_ muscarinic acetylcholine receptor” OR “M_3_ muscarinic acetylcholine receptor” OR “M_4_ muscarinic acetylcholine receptor” OR “M_5_ muscarinic acetylcholine receptor” OR “muscarinic receptor antagonist” OR “muscarinic receptor agonist”) AND (“retina” OR “ocular” OR “RGC” OR “glaucoma” OR “IOP” OR “diabetes” OR “retinal models”)). The search was conducted from 13 March to 28 March 2021 with the following inclusion criteria: all studies, muscarinic acetylcholine receptors in the retina, written in English and published after 1976. In total, the study search resulted in 268 publications. Studies reporting the roles of muscarinic acetylcholine receptors in other organs except for eyes were excluded. Studies in conjunctiva, cornea, iris and lens were excluded. Moreover, expert opinions, abstracts, and letters were excluded. The reference lists of all retrieved articles were reviewed for further identification of potentially relevant studies. 

## 2. Expression and Distribution of mAChRs in the Retina

Based on studies with the labeled radioligands, [^3^H]propylbenzilylcholine mustard ([^3^H]PrBCM), [^3^H]N-methylscopolamine ([^3^H]NMS) and [^3^H]quinuclidinyl benzilate ([^3^H]QNB), a high density of muscarinic binding sites has been demonstrated in rat, bovine and chick retinas, whereas relatively few binding sites have been detected in frog and salamander retinas [37,38,39]. Autoradiographic experiments in embryo and adult chicken retinas revealed specific mAChR binding sites in the inner synaptic retinal layer [40]. In 1985, Polans et al. found a high density of muscarinic binding sites in the inner plexiform layer (IPL) and the outer plexiform layer (OPL) of the salamander retina [41]. In the same year, Hutchins and Hollyfield presented evidence for a population of mAChRs in the human retina, apparently expressed in the IPL, by using the irreversible ligand, [^3^H]PrBCM [42].

Based on autoradiographic experiments, it has been suggested in 1988 that mAChR subtype number and distribution change during retinal development [43]. Later, experiments in the ferret retina suggested that the subtypes, number and distribution of mAChRs changes during retinal synaptogenesis [44]. In the study, mAChR-like immunoreactivity was found at amacrine–amacrine cell contacts by electron microscopy and immunohistochemical techniques [44]. Townes-Anderson and Vogt found that mAChRs in the salamander retina are located on amacrine/ganglion, bipolar, and horizontal cells [45]. In 1988, Moroi-Fetters found that stimulation of muscarinic receptors by the subtype-preferring M_1_ receptor antagonist, pirenzepine, in the rat retina causes phosphoinositide hydrolysis, which indicated that these receptors appear to be of the M_1_ subtype [46].

In 1989, all five mAChR subtypes (M_1_, M_2_, M_3_, M_4_ and M_5_) were identified [47,48]. Molecular cloning techniques provided a new molecular basis to characterize expression, location and physiological function of all five mAChRs [49]. In 1997, McKinnon et al. examined regulation of mAChR expression in the chicken embryonic retina by using immunoblot, immunoprecipitation and solution hybridization analyses [50]. The authors reported that the M_4_ receptor is the main subtype expressed at an early stage in embryonic development, while M_2_ and M_3_ receptor expression increases at a later stage [50]. 

**Figure 1:**
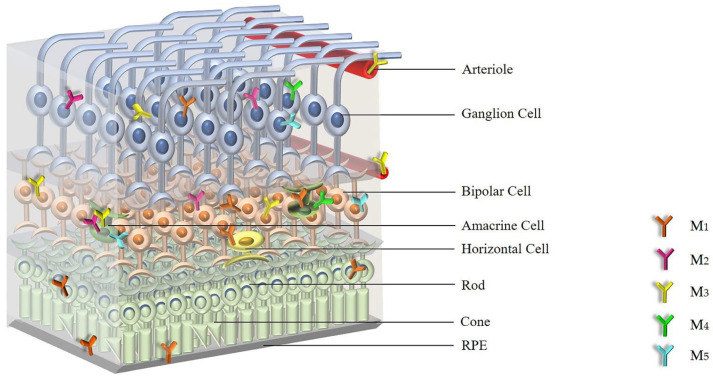
The distribution of individual mAChR subtypes in the retina. Abbreviations: mAChRs: muscarinic acetylcholine receptors; RPE: retinal pigment epithelium; M_1_: M_1_ muscarinic acetylcholine receptor; M_2_: M_2_ muscarinic acetylcholine receptor; M_3_: M_3_ muscarinic acetylcholine receptor; M_4_: M_4_ muscarinic acetylcholine receptor; M_5_: M_5_ muscarinic acetylcholine receptor.

However, the precise anatomical location of mAChRs in the retina remained unknown at that time. One year later, Fischer et al. used purified and subtype-specific antibodies directed against M_2_, M_3_ and M_4_ subtypes to detect receptor immunoreactivity in the retina. The study revealed that in the chick retina the M_2_ receptor was expressed in amacrine and ganglion cells, the M_3_ receptor was expressed in many bipolar cells and small subsets of amacrine cells and the M_4_ receptor was found in amacrine and ganglion cells [51]. In an in vitro and in vivo study, Belmonte et al. demonstrated that retinal Müller glial cells can secrete a factor called MARIA (muscarinic acetylcholine receptor-inducing activity) that can regulate M_2_ expression in vitro and in vivo [52]. Another in vitro study demonstrated the presence of the M_1_ receptor in cultured human retinal pigment epithelium (RPE) at both the mRNA and the protein level [53]. The expression of M_1_ receptor mRNA was also observed in the guinea pig retina, and immunohistochemical findings revealed that the M_1_ receptor was expressed in all layers of the retina [54]. Strang et al. used RT-PCR, Western blot analysis and immunohistochemistry to identify the expression and distribution of mAChR subtypes in the rabbit retina [55]. The authors detected mRNA expression for all five mAChR subtypes in the whole neural retina by RT-PCR and Western blotting, and they confirmed that all five mAChR subtypes were expressed by subpopulations of bipolar, amacrine, and ganglion cells by immunohistochemical analyses [55]. According to a study by Gericke et al. in 2011, only mRNA for the M_3_ receptor was detected in murine retinal arterioles [56]. In contrast, mRNA for all five mAChR subtypes was detected in ophthalmic arteries, but mRNA levels for the odd-numbered subtypes, M_1_, M_3_ and M_5_, were higher than those for the even-numbered subtypes, M_2_ and M_4_ [57]. Figure 1 shows the distribution of mAChRs within the retina. 

Based on the expression studies, all five mAChR subtypes have been detected in the retina with the individual subtypes showing an overlapping expression pattern. 

## 3. Cellular Signaling of mAChRs

Peralta et al. and Bonner et al. first cloned and sequenced human mAChRs, which are encoded by the *CHRM1* to *CHRM5* genes [58,59]. The genes give rise to the five subtypes, M_1_, M_2_, M_3_, M_4_ and M_5_ [3]. The mAChR family belongs to the superfamily of seven-transmembrane receptors, which mediates cellular signal transduction pathways via G-proteins [60]. The M_1_, M_3_ and M_5_ subtypes, which efficiently couple to Gq/11 subtype G proteins, can mobilize phosphoinositides to generate inositol 1, 4, 5-triphosphate (IP3) and 1, 2-diacylglycerol (DAG) via activation of phosphoinositide-specific phospholipase Cβ (PLCβ), leading to an increase in intracellular cytosolic calcium (Ca^2+^) levels and protein kinase C (PKC) activity [61]. This may help to stimulate nitric oxide (NO) production, since neuronal nitric oxide synthase (nNOS) is calcium/calmodulin-dependent [62]. The M_2_ and M_4_ receptors preferentially couple to pertussis toxin-sensitive Gi and Go proteins, causing an inhibition of the cAMP-dependent pathway via suppression of adenylyl cyclase [63]. Furthermore, both Gq/11-, Gi/o-coupled with mAChRs may exert effects through activation of small GTPases, such as Rho and Ras, and downstream effectors, such as phosphoinositide-3 kinases and mitogen-activated protein kinases [60]. 

Muscarinic receptors play an important role in the development of the retina and in processing visual information [64]. For example, mAChRs regulate the function of bipolar cells in the ON/OFF channel, and the input and output of amacrine, bipolar, ganglion and horizontal cells [52,64]. Additionally, Jardon et al. suggested that a cholinergic loop of amacrine cells could be involved in the inhibitory pathway from the ON channel to the OFF channel in the frog retina carrying “light on” and “light off” information from the retina to the brain [65,66]. Muscarinic cholinergic transmission exerts a substantial contribution in the retina [67]. In an in vitro study, the muscarinic antagonist, QNB, enhanced the amplitude of the electroretinogram (ERG) b-wave (a measure of ON bipolar cell activation), and induced moderate vasoconstriction in the cat retina [67]. 

Although mAChRs regulate many important cellular signaling pathways, it is difficult to assign specific functional roles for individual mAChR subtypes in the retina. Jositsch et al. tested the specificity of mAChRs antibodies under different conditions in immunohistochemical labelling on tissue sections by analyzing specimens from respective gene-deficient mice and wild-type mice [68]. The data indicated that immunohistochemical detection of mAChR subtypes in tissue sections is limited to the M_2_ subtype [68]. It has been reported that cells frequently co-express more than one mAChR subtype that increases the difficulty of assigning a functional response to a single receptor subtype [3]. The lack of highly selective pharmacological ligands and antibodies for individual mAChR subtypes has hampered conclusions regarding the physiological role of individual muscarinic receptor subtypes. The development of genetically modified mice devoid of M_1_- to M_5_ receptors helped to circumvent the problem of assigning a specific function to an individual mAChR subtype [69,70,71,72,73,74,75]. M_1_-M_5_ receptor knockout mouse models (M_1_-M_5_R−/−) have also been used in studies of ocular tissues. 

For example, Barathi et al. studied the role of each of the mAChR subtypes in the development of myopia by using M_1_-M_5_R−/− mice [76]. The authors found that M_2_ receptors play a crucial role in myopia development by hindering scleral fibroblast cell proliferation and further scleral remodeling [76]. Based on studies in M_3_R−/− mice, Gericke et al. showed that the M_3_ receptor is responsible for mediating cholinergic responses in retinal arterioles and the ophthalmic artery [56,57,77]. Laspas examined the amount of cells in the retinal ganglion cell (RGC) layer and the amount of axons in the optic nerve in 5-month-old M_1_R−/− and wild-type mice and found no significant difference between both groups [78]. More recently, the same laboratory conducted experiments in 5- and 15-month-old M_1_R-M_5_R−/− mice to examine whether one of the mAChRs and age have an influence on neuron survival in the retina [30]. Based on these studies, the M_1_ receptor was found to be critical for RGC survival in the aging mouse retina [30]. These examples show how genetically modified mice may help to better understand the physiological roles of individual muscarinic receptor subtypes in the retina. 

## 4. Functional Roles of Individual mAChR Subtypes in the Retina

According to their differential coupling to intracellular signaling cascades, the mAChR subtypes have been divided into two subfamilies, the “M_1_-like” mAChR subfamily and the “M_2_-like” mAChR subfamily [79]. The odd-numbered subtypes, M_1_, M_3_, and M_5_, belong to the “M_1_-like” family, which couple to the Gq protein and activate phospholipase C (PLC)-dependent signaling pathways. In neuronal tissue, activation of this signaling cascade increases neuronal excitability through activation of nonspecific cation channels, release of Ca^2+^ from intracellular stores, or inhibition of Ca^2+^-activated K^+^ channels [80]. On the contrary, members of the “M_2_-like” family, the even-numbered M_2_ and M_4_ receptor subtypes, are generally linked to inhibition of adenylyl cyclase activity [79]. Activation of M_2_ and M_4_ receptors decreases neuronal activity via activation of a subset of K^+^ channels, the inhibition of Ca^2+^ channels, or the inhibition of the Ca^2+^ priming of K^+^ channels [55,81,82]. This suggests that the different subtypes may subserve different functions in the retina. 

A plethora of studies demonstrated that the M_1_ muscarinic receptor subtype may be crucial for neuron survival in the retina [30]. For example, M_1_ receptor activation protected retinal neurons from glutamate-induced cytotoxicity [83]. It has been proposed that activation of M_1_ receptors reduces Ca^2+^ influx into the cell and the expression of Bcl-2 and Caspase-3 [62,83,84]. This effect can be blocked by the M_1_-preferring muscarinic receptor antagonist, pirenzepine [84]. Additionally, activation of M_1_ receptors significantly increases the survival of RGCs in vitro [85]. Pereira et al. indicated that the mechanism involved in M_1_ receptor activity and the survival of RGCs is by the release of polypeptides and activation of insulin receptor kinase receptors [85]. An in vitro study demonstrated that activation of mAChRs effectively protects against hypoxia-induced apoptosis in RGCs via modulation of the hypoxia-inducible factor 1-alpha (HIF-1α) pathway [86]. Based on these in vitro data, Laspas et al. conducted a study in 5-month-old M_1_R−/− mice and age-matched wild-type mice to test the neuroprotective role of the M_1_ receptor in vivo. However, the authors found no differences in the number of retinal neurons and the amount of optic nerve axons between M_1_R−/− and wild-type mice [30]. In a more recent study in 2019, Laspas et al. examined the potential role of all five muscarinic receptor subtypes on neuroprotection in the RGC layer in congenic mAChR−/− mice of different age categories [30]. Intriguingly, the authors observed that the lack of the M_1_ receptor was associated with a reduced RGC density in aged mice. Aged M_1_R−/− mice also displayed elevated ROS levels in the RGC layer and increased retinal mRNA expression for the prooxidative NADPH oxidase 2 (NOX2) and reduced mRNA levels for the antioxidative enzymes, superoxide dismutase 1 (SOD1), hemeoxygenase-1 (HO-1) and anti-glutathione peroxidase 1 (GPx1) [30]. The findings provided the first direct evidence that the lack of the M_1_ receptor leads to accelerated RGC loss in mice via changing in the oxidative/antioxidative balance in favor of oxidative in the retina [30]. L-satropane was reported to be effective in preventing retinal neuron damage, which may be attributed to decreasing cell apoptosis and amyloid-β (Aβ) production via activation of M_1_ receptor subtype [87]. Moreover, in an in vivo study, the M_1_ muscarinic receptor was reported to exert protective effects on RGCs via activation of insulin growth factor 1 (IGF-1) and insulin growth factor 1 receptor (IGF-1R) [88]. Additionally, activation of PKC delta was suggested to regulate neurotrophin levels by M_1_ muscarinic receptor activation ultimately leading to an increase in RGCs’ survival in vitro in the retina [89,90]. In 2021, an in vivo study in rats showed that huperzine A lowers intraocular pressure via the M_3_ receptor and exerts neuroprotective effects in the retina by increasing endogenous ACh levels and activating M_1_ receptors and their downstream AKT/MAPK signaling pathways [31]. 

Braga et al. first analyzed the levels of M_3_ receptors in retinal cell cultures treated with 50 ng/mL phorbol 12-myristate 13-acetate (PMA, a PKC activator) for 48 h. PMA induced a marked increase in M_3_ receptor levels [91]. Based on pharmacological studies employing muscarinic subtype-preferring antagonists, Borda et al. observed that carbachol can stimulate NOS activity and increase the expression of nNOS and iNOS mRNA in the rat retina via activation of M_1_/M_3_ receptor subtypes [92,93]. 

The expression of nicotinic AChR (nAChR: α3, α4, α6, α7, β2 and β4 nAChR subunits) and/or mAChR by amacrine and ganglion cells has been described in retinas of Rhesus monkeys and rabbits [94,95,96]. Retinal nAChRs mediate visual processing and may have effects on refractive development and ocular neovascularization [94]. In the retina, there was an overlap in the expression patterns of M_1_, M_4_ and M_5_ muscarinic receptors with those of non-α7 and α7 nAChRs in presumptive amacrine and ganglion cells [97]. Strang et al. suggest that the determining the role of mAChRs in retinal processing is complicated by the concomitant expression of nAChRs by the same cells [55]. A study in α7 nAChR−/− mice demonstrated that M_2_ and M_4_ mAChR subtype transcripts were significantly upregulated in the RGC layer [98]. 

There are not many studies on the functional role of M_2_ receptors in the retina. Several pieces of evidence suggest that activation of M_2_ and M_4_ receptors is involved in visual processing [55]. The M_2_ receptor has been reported to increase Ca^2+^ influx, exclusively due to Ca^2+^ mobilization from intracellular stores [99]. The M_2_ muscarinic receptor was shown to inhibit adenylyl cyclase activity and to activate inwardly rectifying potassium (K^+^) channels [100]. However, these responses can be rapidly attenuated by receptor desensitization [100,101]. Antal et al. found that activation of M_2_ receptors regulates feedforward inhibition following activation of RGC synapses in a manner that is strongly dependent on the number of activated RGCs [102]. Cimini et al. indicated that the production of NO in response to M_2_ muscarinic receptor activation may lead to an increase in cGMP, which can modulate the mutual interactions of acetylcholine-glycine-gamma-aminobutyric acid (GABA) in the inner retina [103]. 

The M_4_ receptor exerts a direct inhibitory control on dopamine D_1_-like receptor signaling [104]. In a rat glaucoma model, Almasieh et al. demonstrated that activation of M_1_ and M_4_ receptors promotes RGCs’ survival [15]. Moreover, the partially selective M_1_/M_4_ muscarinic antagonist, pirenzepine, was reported to be successful in preventing myopia progression in animal models [105]. The M_4_-selective antagonist, himbacine, could also prevent myopia in chicken by daily intravitreal injections [106]. 

All these studies provide evidence that individual mAChRs exert specific functional roles in the retina, which offers new therapeutic perspectives for mAChR ligands. A variety of studies reported associations of endothelial NOS (eNOS) genetic polymorphism or adrenergic receptor gene polymorphisms with retinal diseases [107,108]. Additionally, mAChR subtype gene polymorphisms have been reported [109]. Unfortunately, studies on mAChR genetic polymorphisms with respect to retinal diseases have not been reported so far [109]. Such studies would be appreciated to shed some light on the role of mAChR in the development of specific retinal diseases. 

## 5. Strategies to Target Individual mAChR Subtypes 

Individual mAChR subtypes are novel targets for the treatment of various diseases including Alzheimer’s disease, Parkinson’s disease, type 2 diabetes, schizophrenia and glaucoma [15,30,110,111,112]. The first-generation muscarinic agonist, pilocarpine, was initially used as a topical glaucoma therapy in the late 1800s and approved by the United States Food and Drug Administration (FDA) in 1974 [113]. Although pilocarpine has been used as an ocular hypotensive agent for 40 years, it causes ocular side effects due to poor selective pharmacokinetic properties. Therefore, clinical approaches are required that modulate individual mAChR subtype activity with high selectivity. However, there are still few ophthalmological studies that focus on subtype-selective mAChR ligands. For example, several randomized double-blind, placebo-controlled studies tested the impact of cevimeline, a specific agonist of the M_3_ muscarinic receptor, on dry eye symptoms in patients with Sjögren’s syndrome and most of them suggested some beneficial effects [114,115,116,117].

Due to high sequence conservation in the orthosteric binding site of mAChR subtypes, it has been difficult to develop mAChR ligands with high subtype selectivity [118]. Subtype-preferring M_1_ agonists used for the treatment of central nervous system (CNS) disorders have been reported in the patent or primary literature. However, subsequent studies indicated that previous orthosteric agonists are not highly selective when evaluated across multiple systems [119]. Moreover, previous studies trying to develop highly selective ligands for individual mAChR subtypes have failed because of the difficulty of developing compounds that are truly subtype-selective [119]. In consequence, many researchers are now focusing on developing allosteric activators of mAChRs including both positive allosteric modulators (PAMs) and allosteric agonists, which offer new opportunities to target specific mAChR subtypes for therapeutic purposes [120]. In the treatment of Alzheimer’s disease and other CNS disorders, several novel selective M_1_ agonists and allosteric potentiators have been identified, providing important new tools to evaluate the potential utility of selective activators of the M_1_ receptor [121]. For example, BPB and 77-LH-28-1 have been reported to exert highly selective agonist activity for the M_1_ receptor [119,122,123]. Lebois et al. have discovered a novel highly selective M_1_ allosteric agonist VU0357017, with a potentially novel allosteric binding site in the third extracellular loop of the M_1_ receptor [124]. The novel PAMs for M_1_, VU0090157, VU0029767 and benzyl quinolone carboxylic acid (BQCA), compete for binding at the orthosteric ACh-binding site, but have no direct agonist activity. However, they induce a robust leftward shift of the concentration-response relationship of ACh at activating the M_1_ receptor [121]. The highly selective PAMs for the M_4_ receptor, VU0010010, VU0152099, VU0152100 and LY2033298, were reported to be an important breakthrough for selective activation of the M_4_ receptor exerting no activity at any other mAChR subtype [124]. By novel microwave-assisted chemistry in in vitro and in vivo probe projects, Weaver et al. described the discovery and development of the first highly selective M_1_ antagonist, VU0255035, which was shown to be active in vivo and penetrated the blood-brain-barrier [118]. 

Apart from the well-known therapeutic applications for CNS diseases, mAChRs subtype ligands may find potential applications in ophthalmology, such as in the field of retinal neuroprotection [125,126,127]. 

## 6. Conclusions

All five mAChR subtypes, M_1_ through M_5_, were found to be expressed in the retina. Experimental studies over the past decade were focusing on the biology, pharmacology and structure of mAChRs. Knockout animal models have provided clues to the specific functions of mAChR subtypes in various physiological and pathophysiological processes of the retina. The M_1_ receptor is suggested to be involved in retinal neuron survival and, therefore, appears to be a promising therapeutic target. Studies of mAChR genetic polymorphisms focusing on retinal diseases as well as studies employing genetically modified animal models and new mAChR ligands with high subtype selectivity will be helpful to shed more light on the physiological and pathophysiological role of individual muscarinic receptor subtypes in the retina. New pharmacologic compounds with high selectivity for individual mAChR subtypes have already been studied in CNS disorders and may also offer attractive tools to treat retinal diseases.
